# GHGKHKNK Octapeptide (P-5m) Inhibits Metastasis of HCCLM3 Cell Lines via Regulation of MMP-2 Expression in *in Vitro* and *in Vivo* Studies

**DOI:** 10.3390/molecules17021357

**Published:** 2012-02-02

**Authors:** Xiao Han, Dong-Mei Yan, Xiang-Feng Zhao, Matsuura Hiroshi, Wei-Guang Ding, Peng Li, Shuang Jiang, Bai-Rong Du, Pei-Ge Du, Xun Zhu

**Affiliations:** 1 Department of Immunology, Norman Bethune College of Medicine, Jilin University, Changchun 130021, China; Email: hanxiaorumeng@126.com (X.H.); yxn345@sohu.com (D.-M.Y.); zxf184@163.com (X.-F.Z.); frank0414@163.com (P.L.); dubairong@yahoo.com.cn (B.-R.D.); 2 Department of Microbial and Biochemical Pharmacy, College of Pharmaceutical Science, Beihua University, Jilin 132001, China; Email: jiangshuang_2000@163.com; 3 Department of Physiology, Shiga University of Medical Science, Otsu, Shiga 520-2192, Japan; Email: matuurah@belle.shiga-med.ac.jp (M.H.); ding@belle.shiga-med.ac.jp (W.-G.D.)

**Keywords:** GHGKHKNK octapeptide, hepatocellular carcinoma, MMP-2, HCCLM3, cleaved high molecular weight kininogen

## Abstract

P-5m, an octapeptide derived from domain 5 of HKa, was initially found to inhibit the invasion and migration of melanoma cells. The high metastatic potential of melanoma cells was prevented by the HGK motif in the P-5m peptide *in vitro* and in an experimental lung metastasis model, suggesting that P-5m may play an important role in the regulation of tumor metastasis. The aim of this study was to measure the effect of P-5m on tumor metastasis of human hepatocarcinoma cell line (HCCLM3) *in vitro* and *in vivo* in a nude mouse model of hepatocellular carcinoma (HCC), and detect the mechanisms involved in P-5m-induced anti-metastasis. By gelatin zymography, matrix metallo-proteinases 2 (MMP-2) activity in HCCLM3 was dramatically diminished by P-5m peptide. In addition, the migration and metastasis of HCCLM3 cells was also inhibited by the peptide *in vitro*. In an orthotopic model of HCC in nude mice, P-5m treatment effectively reduced the lung metastasis as well as the expression of MMP-2 in the tumor tissues. Overall, these observations indicate an important role for P-5m peptide in HCC invasion and metastasis, at least partially through modulation MMP-2 expression. These data suggests that P-5m may have therapeutic potential in metastatic human hepatocarcinoma.

## 1. Introduction

Hepatocellular carcinoma (HCC) is now the seventh most frequent malignancy and the third leading cause of cancer-related deaths in the World [1]. HCC is characterized by rapid progression, early metastasis and frequent recurrence. Although improvements have been made in diagnosis and treatment of HCC, it remains an aggressive cancer with a poor prognosis. Tissue invasion and metastasis are primary cause of the mortality of HCC patients. Therefore, many efforts have been made to find a more efficient treatment to inhibit tumor metastasis. The process of metastasis includes separation of tumor cells from the original niche, invasion of underlying basal lamina, entry to cardiovascular or lymphatic circulation and formation of secondary lesions [2,3]. Degradation and remodeling of extracellular matrix (ECM) are necessary steps in local invasion. Excess ECM degradation is one of the hallmarks of tumor migration. Matrix metallo-proteinases (MMPs), a superfamily of zinc-dependent endopeptidases, play a crucial role in ECM degradation. MMP-2 and MMP-9 degrade most of the ECM components of basal membrane and type IV collagen. Increased expression and activity of MMP-2 has been well characterized in human HCC [4]. Basal membrane degradation correlated positively with MMP-2 and MMP-9 proteolytic activity [4]. Therefore, the inhibition of migration or invasion mediated by MMPs might represent a strategy for preventing hepatocarcinoma metastasis [5]. 

High molecular weight kininogen (HK) is a multifunctional plasma glycoprotein that plays important roles in pathophysiological processes such as fibrinolysis, thrombosis, inflammation and angiogenesis [6,7]. Single-chain HK, 120 kDa, composed of six domains (D1 to D6), is complexed in plasma with prekallikrein [8]. Most plasma prekallikrein circulates bound to HK and upon binding to the endothelial cell surface via HK is converted to kallikrein by prolylcarboxypeptidase (PrCP). After hydrolytic cleavage by kallikrein, HK releases bradykinin from domain 4 (D4), generating two-chain high molecular weight kininogen (HKa) (D1-D3 and D5-D6) linked through a single disulﬁde bond [9]. The transition from uncleaved kininogen to HKa involves extensive conformational changes resulting in a greater exposure of the Domain 5 (D5) region, and such domain rearrangements augment anti-adhesive properties. Studies from our laboratory [7,10] and others [6,11,12] have demonstrated that D5 has significant anti-adhesive and anti-invasion properties towards tumor cells, although the mechanism by which D5 inhibits adhesion and invasion remains controversial. Notably, D5 is a His-Gly-Lys (HGK) rich region (residues 402–502), the contents of His, Gly and Lys reach 24.8%, 22.7% and 14.9%, respectively. Various data indicat that anti-cell adhesion and invasion activities depend on the amino acid sequence in D5. Previous reports have indicated that His-Gly-Lys-rich subdomains, such as residues H^479^-K^493^ [11], residues H^475^-K^502^ [6] and residues G^486^-G^496^ [12], were responsible for inhibition of cell migration. Kawasaki *et al.* [10] first reported that an octapeptide derived from D5, namely P-5m (Gly-His-Gly-Lys-His-Lys-Asn-Lys, residues 484–491), inhibited vitronectin-mediated invasion and migration of breast cancer cells and melanoma cells *in vitro*. In addition, P-5m peptide reduced the high metastatic potential of melanoma cells *in vivo*. P-5m is the shortest sequence reported within D5 which has anti-metastatic activity comparable to the full-length D5 sequence. In contrast, although D5 has been demonstrated to have potent anti-angiogenic activity, P-5m had no inhibitory effect on angiogenesis.

In recent years, increasing attention has been paid to synthetic antitumor peptides. Modern chemotherapy based on synthetic oligopeptides has the potential to provide an effective anti-metastasis treatment for cancer patients whilst minimizing severe side-effects [13,14,15,16]. Although it is quite clear that P-5m may inhibit the invasion of various cancer cells *in vitro* and melanoma pulmonary metastasis in animal models *in vivo*, the precise impact of P-5m on metastasis of human cancer cells in an *in vivo* model is still uncertain. In the present study, we aimed to measure the effect of P-5m on tumor metastasis of a HCCLM3 human hepatocarcinoma cell line and *in vivo* in a nude mouse model of hepatocellular carcinoma and to detect the underlying mechanisms of any inhibitory effects. 

## 2. Results and Discussion

### 2.1. Effect of P-5m on the Activation of MMP-2 in HCCLM3 Cells

**Figure 1:**
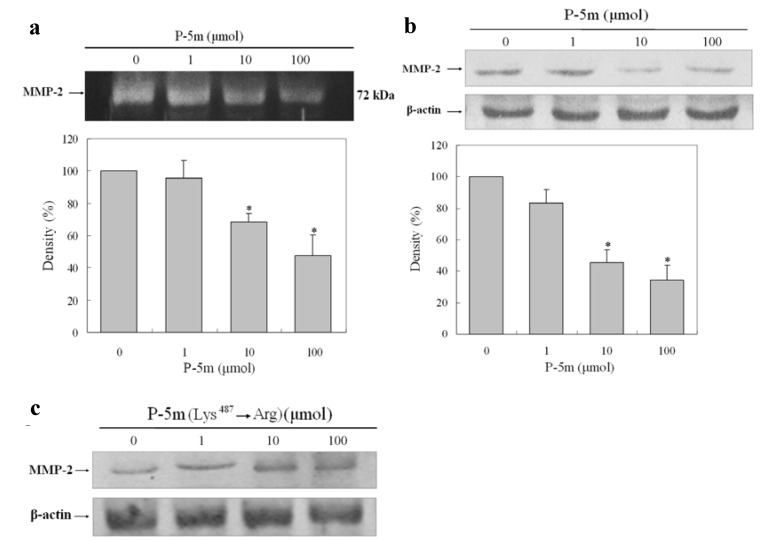
Effect of P-5m and P-5m (Lys^487^→Arg) mutant on MMP-2 secretion in HCCLM3 cells. (**a**) HCCLM3 cells were cultured with or without P-5m for 36 h. The conditioned media were analyzed by zymography. (**b**,**c**) HCCLM3 cells were cultured with or without P-5m/P-5m mutant for 36 h. The protein levels of MMP-2 from whole-cell lysates were analyzed by Western blotting with β-Actin as a control. Bars represent mean ± SD of three independent experiments. * *P* < 0.05.

**Figure 2:**
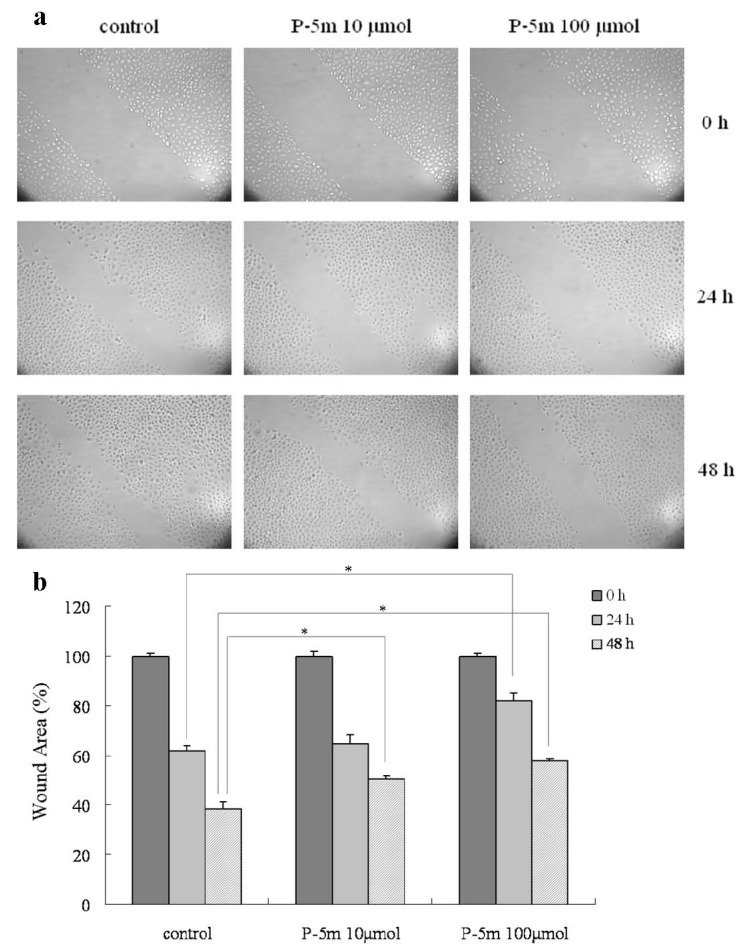
Effects of P-5m on migration in HCCLM3 cells. Cell migration was analyzed by wound healing assay. (**a**) Cells were cultured with P-5m (0, 10 and 100 μmol) for 48 h. Photographs were taken 0, 24 and 48 h after a wound was generated. (**b**) Cell migration was analyzed by NIH Image software (Image J). The results were expressed as percentages of the mean of wound area at 0, 24 and 48 h. The quantitative data are presented as mean ± SD of three independent experiments. * *P* < 0.05, compared with the respective untreated group.

**Figure 3:**
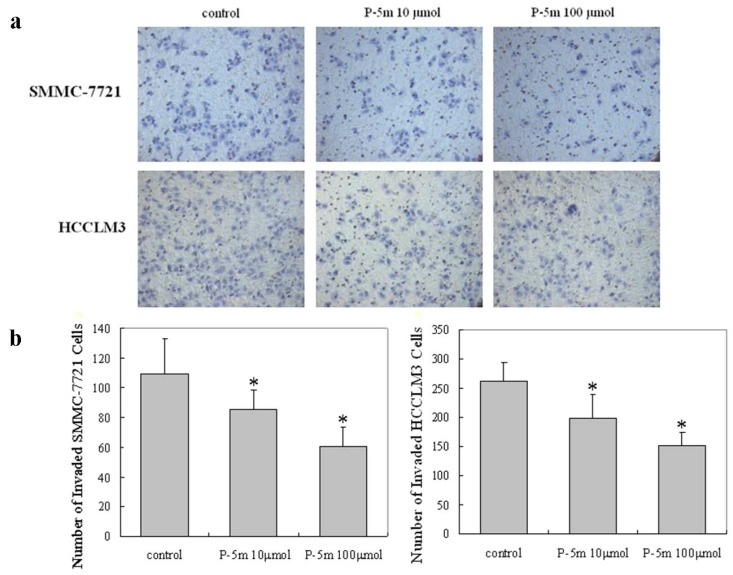
Effects of P-5m on cell invasion by SMMC-7721 and HCCLM3 cells. (**a**) SMMC-7721 or HCCLM3 cells were treated with various concentrations of P-5m (0, 10 and 100 μmol) for 48 h. Cells in the lower chamber were then stained and quantified. (**b**) For each plate, a representative number of invaded cells were counted under the microscope and averaged in five random fields at ×400 magnification. Bars represent mean ± SD of these averages from triplicate plates. * *P* < 0.05, compared with the control.

**Figure 4:**
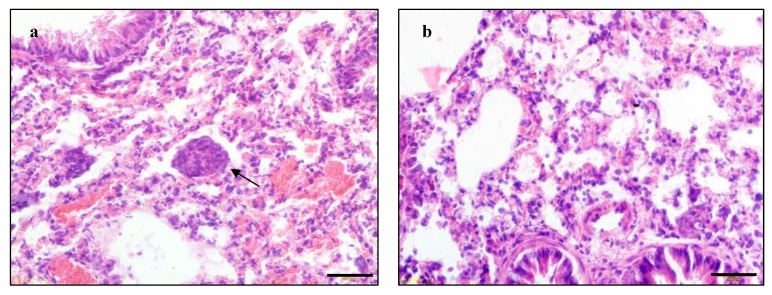
The representative example of pulmonary metastasis (**a**, arrow indicating a metastatic tumor nodule) and non-metastasis (**b**) in the HCCLM3 xenograft model, by H&E staining. The black bar represents 100 µm.

**Figure 5:**
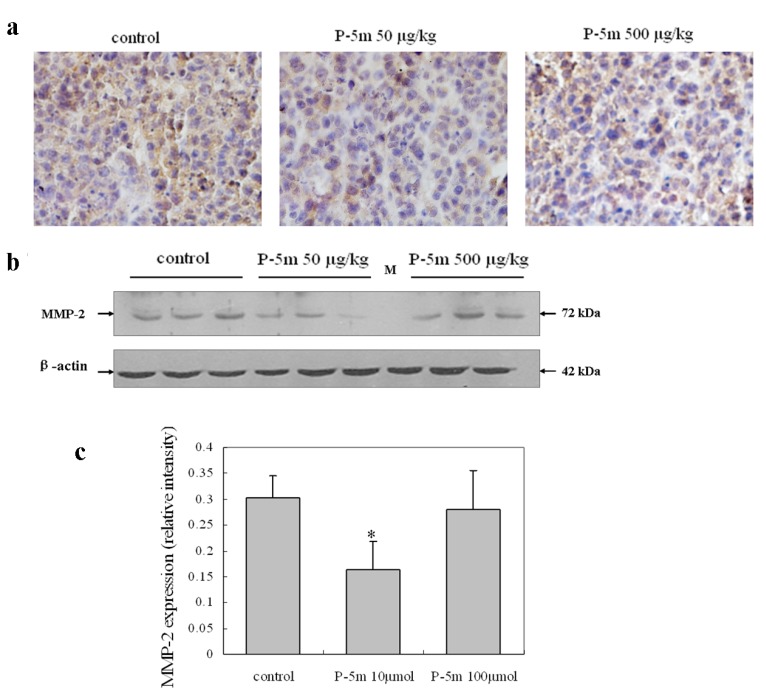
Effects of P-5m on MMP-2 expression in the HCCLM3 xenograft model. (**a**) Immunostaining showing reduced MMP-2 expression in the 50 μg/kg P-5m treated group compared with the control group. (**b**) Western blots showing decreased MMP-2 expression in the 50 μg/kg P-5m treated group compared with the control group. (**c**) Quantitative data, represented as mean ± SD of three tumor nodules. * *P* < 0.05, compared with the respective untreated group.

Increased expression and activity of MMP-2 has been well characterized in human HCC [4]. To determine whether P-5m inhibits the MMP-2 activity of HCCLM3 cells, the cancer cells were treated with various concentration of P-5m in serum-free media. Because the expression of MMP-2 in HCCLM3 cells peaked at 36 h after incubation, this time-point was selected as optimal for further experiments. The conditioned media were analyzed by gelatin zymography. As shown in Figure 1a, P-5m treatment reduced MMP-2 release in a dose-dependent manner. Densitometric analysis of the zymograms showed reductions of 95.6%, 68.2% and 47.6% compared to control at 1, 10, and 100 μmol of P-5m, respectively. In addition, the cell samples were also lysed to detect MMP-2 expression by western blotting (Figure 1b). Densitometric analysis of the MMP-2 levels in western blots showed reductions of 84.2%, 46.4% and 33.8% compared to control at 1, 10, and 100 μmol of P-5m, respectively. Thus, changes in production of MMP-2 measured intracellularly and extracellularly by western blotting and gelatin zymography respectively, were concordant. Kawasaki *et al.* [10] designed previously a point-mutated peptide with a substitution of Lys^487^ to Arg in P-5m. This mutant peptide did not inhibit the adhesion and invasion of melanoma cells in adhesion and invasion assays. We also employed this mutant peptide in the MMP-2 western blotting assay to confirm the specificity of P-5m treatment in our study. As shown in Figure 1c, the mutant had no inhibitory function on MMP-2 levels produced by HCCLM3 cell lines.

Generally, MMP-2 is constitutively expressed and over secreted in highly metastatic tumors, whereas MMP-9 is induced in response to stimulattion by different protein factors using different intracellular-signaling pathways [17]. The impact of P-5m on the MMP-9 activity in this study was inconclusive, since an extremely low level of MMP-9 was detected in HCCLM3 cells (data not shown).

### 2.2. P-5m Inhibits the Migration and Invasion of HCCLM3 Cells

Cancer cell migration and invasion are two important steps in cancer metastasis. We examined the effects of P-5m peptide on cell migration and invasion by tumor cells. An *in vitro* migration assay was used to investigate the inhibitory effect of P-5m. As shown in Figure 2a, the numbers of HCCLM3 cells in the wounded area were significantly elevated at 24 h and 48 h as compared with controls. The quantitative assessment (Figure 2b) showed that treatment with 10 and 100 μmol of P-5m inhibited cell migration by 5% and 33%, respectively, at 24 h. At 48 h, 10 and 100 μmol of P-5m inhibited cell migration by 30% and 49%, respectively.

Furthermore, the effect of P-5m on invasion by HCCLM3 cells and cells of an additional human hepatocarcinoma cell line (SMMC-7721) was determined in a transwell chamber and basement membrane matrigel invasion assay. Quantitative data derived from three independent experiments shows that P-5m effectively inhibited the cell invasion in this assay (Figure 3). Reductions were concentration-dependent: at the at the lower dose (10 μmol) of P-5m, reductions were 14.3% and 16.8%, for SMMC-7721 and HCCLM3 cells, respectively; at the higher dose (100 μmol) of P-5m, reductions were 14.3% and 16.8%, for SMMC-7721 and HCCLM3 cells, respectively.

### 2.3. P-5m Suppresses Pulmonary Metastasis of Hepatocellular Carcinoma *in Vivo*

We further examined the therapeutic efficacy of P-5m against tumor metastasis in an orthotopic hepatocellular carcinoma animal model. HCCLM3 has been reported to undergo pulmonary metastasis after subcutaneous implantation into nude mice [18]. In the present study, we used HCCLM3 to establish a nude mouse orthotopic implantation model of human hepatocellular carcinoma which may more effectively mimic natural human biologic behavior. At first, fresh tumor tissues were transplanted into the left lobe of the liver of nude mice. On the third day, the mice were divided randomly into four experimental treatment groups. During the experiment, 5-Fu-treated mice showed serious sighs of toxicity. Since only three mice survived in the 5-Fu group at the end of the study, we did not perform statistical analysis on this group. Death did not occur in any of the other three groups during the observation period. All of the mice were sacrificed at 30 days. There was no noticeable difference in hepatic tumor size between untreated mice and those treated with either dose of P-5m by macroscopic observation. However, the HCCLM3 implanted tumors showed infiltrative growth patterns. After P-5m treatment, most tumor nodules exhibited a growth pattern characterized by a clear boundary between the tumor and the normal liver tissue. The metastasis nodules in the lung were counted and verified by HE staining (Figure 4). 

Single or fused small metastatic nodules were found in the lungs, and no nodules were found in other organs examined. There was significant suppression of pulmonary metastasis in animals treated with 50 μg/kg P-5m therapy compared with the control group (*p* < 0.05, Table 1). Although 500 μg/kg P-5m treatment caused some reduction in lung metastasis, this was not statistically significant (*p* > 0.05, Table 1).

**Table 1:** Effect of P-5m peptide on lung metastasis in orthotopic model of HCC in nude mice.

Treatment group	Mice-bearing pulmonary metastasis/total mice	Percentage
Control	6/8	75%
P-5m 50μg/kg	2/8	25%
P-5m 500μg/kg	4/8	50%

* *P* < 0.05, Fisher’s Exact test versus control group. All statistical tests were two-sided.

Thus, the 50 µg/kg P-5m appeared to be more effective than the 500 µg/kg dose. However, we also previously observed this phenomenon in another mice hepatoma ascites tumor model established by our laboratory (data not shown). The reasons behind this phenomenon are unclear, although one possibility is that the receptor is internalized at the higher dose: most biologically active peptides have receptors on the plasma membrane and binding to ligands will trigger receptor internalization and downregulation, although the fate of these receptors within the cells differs [19,20]. Thus the presence of exogenous molecules in lysosomes is associated with degradation, in the Golgi apparatus with synthesis or recycling, and in nucleus with gene expression. Kawasaki *et al.* [10] detected a 95 kDa D5-binding protein on breast cancer cells, which was detached from D5 by P-5m. They speculated that the 95 kDa protein is the receptor of P-5m on the cell membrane and hypothesized that excess P-5m peptide may induce extensive receptor internalization and downregulation, with partial degradation in lysosomes. Although similar mechanisms may account for our observation, this will require further investigation.

### 2.4. P-5m Inhibits MMP-2 Expression in Orthotopic Hepatoma Animal Model

Immunohistochemical analysis showed that MMP-2 was present in all tumor samples from primary nodules, although differences in staining intensity were evident between groups (Figure 5). MMP-2 staining seemed to be more abundant in control group mice than either P-5m groups (Figure 5a). Although MMP-2 appeared to show a largely intracellular distribution, it was also present in extracellular matrix. Western blotting further confirmed that the 50 μg/kg P-5m dose, but not the 500 μg/kg dose, significantly decreased expression of MMP-2 by 46.7% in hepatoma tissue from mice (Figure 5b,c). This suggests that the P-5m peptide suppressed metastasis, at least in part through down-regulation of MMP-2, which is a crucial molecule in tumor metastasis.

### 2.5. Regulation of Tumor-Related Genes *in Vivo* by Treatment with P-5m

To study the molecular mechanisms through which P-5m regulates hepatocellular carcinoma cells motility, we compared expression of several genes in P-5m (50 μg/kg) treated and control mich (Table 2). The quantitative real-time PCR used contains 24 unique genes related to degradation of ECM, adhesion molecules, metastasis-related markers, apoptosis-related factors, cell cycle regulators, immune-regulating factors together with other genes. All genes represented by the real-time PCR showed a single peak on the melting curve characteristic for the specific products. GAPDH was used for normalization. At greater than 3-fold up-regulation and less than 0.7-fold down-regulation in mRNA expression after treatment with 50 μg/kg P-5m relative to control mice, was considered to be significant. As shown in Table 2, six genes were up-regulated in xenografts, namely TIMP-1, TIMP-2, CASP-3, MTSS-1, TGF-β1 and IL-6. The up-regulation of the former five genes was approximately 3-fold, but expression of IL-6 was increased 13-fold relative to the control. Decreased expression of MMP-2 in tumors was paralleled by the elevated mRNA levels of metalloproteinase inhibitor TIMP-1 and TIMP-2. These data further supported our hypothesis that the effect of P-5m on HCC is mediated through regulating expression of MMP-2. The members of the caspase family play an essential function in apoptosis. CASP-3 is an effector caspase that functions as a central regulator of apoptosis [21]. Although MTSS-1 is considered to function as a metastatic suppressor in some cancers, the relevance of its down-regulation to tumor metastasis has not been confirmed [22]. TGF-β1 and IL-6 have dual roles HCC pathogenesis, acting as tumor suppressors or promoters at different stages [23]. It is clear that further studies need to be conducted to understand the regulatory mechanisms of P-5m.

**Table 2:** Modulated genes relative to control after treatment of xenografts with P-5m.

Name of gene	Symbol	GenBank ID	Primer	Regulation fold	
**Proteinases involved in the degradation of ECM**				
Matrix metallopeptidase 2	MMP-2	NM_001127891.1	5'-ATTTGGCGGACTGTGACG-3'	0.67	
			5'-AGCCTTCTCCTCCTGTGGG-3'		
TIMP metallopeptidase inhibitor 1	TIMP-1	NM_003254.2	5'-CCGCAGCGAGGAGTTTCT-3'	3.17	
			5'-ACAGCCAACAGTGTAGGTCTT-3'		
TIMP metallopeptidase inhibitor 2	TIMP-2	NM_003255.4	5'-CTGGACGTTGGAGGAAAGAA-3'	3.84	
			5'-CTTCTTCTGGGTGGTGCTCA-3'		
**Adhesion molecules**					
CD44 molecule (Indian blood group)	CD44	NM_000610	5'-ATCTTGGCATCCCTCTTGG-3'	1.42	
			5'-GTCCACTTGGCTTTCTGTCC-3'		
Cadherin 1, type 1, E-cadherin (epithelial)	CDH-1	NM_004360.3	GAATCCAAAGCCTCAGGTCA-3'	2.11	
			5'-TCAAAATGATAGATTCTTGGGTT-3'		
Vascular cell adhesion molecule 1	VCAM-1	NM_001078.3	5'-TGCCGAGCTAAATTACACATTG-3'	0.91	
			5'-ACAGAGCCACCTTCTTGCAG-3'		
**Metastasis-related markers**					
Non-metastatic cells 1, protein (NM23A) expressed in (NME1)	NM23	NM_198175	5'-GGATTCCGCCTTGTTGGT-3'	2.17	
			5'-GGCCCTGAGTGCATGTATTT-3'		
Metastasis associated 1	MTA-1	NM_004689.3	5'-AGACCCTGCTGGCAGATAAAG-3'	0.73	
			5'-GCTTGTCTGTGAGTGGGTTGT-3'		
Metastasis suppressor 1	MTSS-1	NM_014751.4	5'-GAAGAACGTGGCCGATTC-3'	3.74	
			5'-GAGGGCAGTTTGTGAGGGTC-3'		
**Apoptosis-related factors**					
Cysteine protease CPP32 isoform alpha	CASP-3	NM_004346	5'-CTGGTTTTCGGTGGGTGT-3'	3.44	
			5'-CAGTGTTCTCCATGGATACCTTT-3'		
B-cell CLL/lymphoma 2	BCL-2	NM_000633.2	5'-TGTCCCTTTGACCTTGTTTCT-3'	1.27	
			5'-TCATTTGCCATCTGGATTTT-3'		
**Cell cycle regulators**					
Cyclin-dependent kinase 4	CDK-4	NM_000075	5'-ACCTGTGGACATGTGGAGTG-3'	0.94	
			5'-ACATCTCGAGGCCAGTCATC-3'		
Cyclin D1	CCND-1	NM_053056	5'-TGTCCTACTACCGCCTCACAC-3'	1.25	
			5'-TTGGGGTCCATGTTCTGC-3'		
Cyclin-dependent kinase inhibitor 1A (p21)	CDKN-1A	NM_000389	5'-GGCAGACCAGCATGACAGAT-3'	1.13	
			5'-AGATGTAGAGCGGGCCTTTG-3'		
Cyclin-dependent kinase inhibitor 2A ( p16)	CDKN2A	NM_000077.4	5'-CATTCATGTGGGCATTTCTTG-3'	1.94	
			5'-TTTGGTTCTGCCATTTGCTA-3'		
**Immune-regulating factors**					
Tumor necrosis factor alpha	TNF-α	NM_000594	5'-CAGCCTCTTCTCCTTCCTGAT-3'	2.12	
			5'-GCCAGAGGGCTGATTAGAGA-3'		
Interleukin 6	IL-6	NM_000600	5'-GATGAGTACAAAAGTCCTGATCC-3'	13.6	
			5'-CTGCAGCCACTGGTTCTGT-3'		
Nitric oxide synthase 2, inducible	NOS-2A	NM_000625	5'-TGCCAAGCTGAAATTGAATG-3'	1.33	
			5'-CCACCCTGTCCTTCTTCG-3'		
Macrophage stimulating 1 receptor	MST-1R	NM_002447.2	5'-CTACATCAACTCCCACATCACC-3'	2.19	
			5'-ATTCAGGAAGGCGGCACA-3'		
Interferon gamma	IFNG	NM_000619.2	5'-CCAACGCAAAGCAATACATG-3'	2.86	
			5'-GCAGGACAACCATTACTGGG-3'		
Major histocompatibility complex, class I, E	HLA-E	NM_005516.5	5'-GCGCGGCTACTACAATCAGA-3'	2.44	
			5'-GGGTGAGATAATCCTTGCCG-3'		
**Others**					
Vascular endothelial growth factor A	VEGFA	NM_001025366	5'-GGCAGAATCATCACGAAGTG-3'	1.45	
			5'-CGATCTCATCAGGGTACTCCT-3'		
Transforming growth factor, beta 1	TGF-β1	NM_000660	5'-GCACGTGGAGCTGTACCA-3'	3.43	
			5'-AAGATAACCACTCTGGCGAGTC-3'		
V-erb-b2 erythroblastic leukemia viral oncogene homolog 2	ERBB-2	NM_001005862.1	5'-TGGAGACCCGCTGAACAATA-3'	0.95	
			5'-GGTTCCGCTGGATCAAGAC-3'		
**House-keeping gene**					
Glyceraldehyde-3-phosphate dehydrogenase	GAPDH	NM_002046	5'-GTGGACCTGACCTGCCGTCT-3'	-	
			5'-GGAGGAGTGGGTGTCGCTGT-3'		

## 3. Experimental

### 3.1. Peptide Synthesis

P-5m Peptide (GHGKHKNK) and P-5m mutant (GHGRHKNK) was synthesized in our laboratory by standard Fmoc solid-phase strategies as previously described [24]. The crude peptide was puriﬁed by reverse-phase HPLC (>98% purity). The molecular weights were identiﬁed by ESI-MS (Agilent Technologies Inc.).

### 3.2. Cell Lines and Animals

The HCCLM3 (human hepatocarcinoma cell line) was obtained from the China Center for Type Culture Collection (CCTCC, Wuhan, China). The SMMC-7721 cell line was maintained in our laboratory. Cells were grown in RPMI-1640 medium supplemented with 10% fetal bovine serum, and incubated at 37 °C in humidified 5% CO_2_. Cells were passaged when they reached 70–80% conﬂuence. The survival rate of cells after isolation was greater than 95% as measured by trypan blue exclusion. 

Female athymic BALB/c nude mice (6 weeks of age) were purchased from the Shanghai Laboratory Animal Center Co. Ltd. (Shanghai, China) and maintained in a pathogen-free animal facility at the Laboratory Animal Research Centre of Jilin University. All experiments were performed with humane care, and were approved by the Animal Care and Use Committee at Jilin University. 

### 3.3. Gelatin Zymography

Gelatin zymography was employed to measure the activities of MMP-2 and MMP-9 as previously described by Birkedal-Hansen *et al.* [25]. Briefly, HCCLM3 cells were seeded (2 × 10^5^/well) in 6-well plates in RPMI-1640 medium with 10% FBS and grown to confluence. Cells were then treated with P-5m (0, 1, 10 and 100 μmol) in serum-free medium for 36 h. For zymography assays, in order to ensure a consistent amount of total protein loaded, the numbers of cells were counted after the supernatant was removed and different volumes of supernatant loaded onto the gel according this cell number. The samples were separated by electrophoresis on 10% SDS-PAGE containing 0.1% gelatin. After electrophoresis, gels were washed with 2.5% Triton X-100 and subsequently incubated in enzyme buffer at 37 °C overnight. The gels were stained with Coomassie blue stain for 30 min and bands corresponding to activity were visualized. Each experiment was carried out in triplicate.

### 3.4. Wound Healing Assay

Cell migration assay was performed as previously described [26]. Briefly, HCCLM3 cells were grown to 90% confluence in a 6-well plate. A wound was created by scratching cells with a sterile 200 μL pipette tip. The plates were washed twice with PBS and then replaced with complete RPMI-1640 medium. Cells were treated with P-5m (0, 10 and 100 μmol) and incubated for 48 h. Photos of the wound were taken at 0, 24 and 48 h, respectively. The level of cell migration was analyzed with NIH Image software (Image J). The results were expressed as a mean percentage of each control (the mean of wound area at stage 0 h).

### 3.5. Invasion Assay

A Boyden chamber invasion assay was used to measure the ability of HCCLM3 cells to pass through filters coated with Matrigel (BD Biosciences, San Jose, CA, USA). Matrigel basement membrane matrix was diluted to 200 μg/mL in cold serum-free medium and used to coat the upper side of 8 μm pore transwell ﬁlters (BD Biosciences). Before the invasion assay, HCCLM3 cells were treated with various concentrations of P-5m peptide for 48 h, detached from the culture plates and resuspended in serum-free RPMI-1640 medium (1 × 10^5^ cells/200 μL). The cells were then seeded in the upper chamber with a serum-containing medium (500 μL) simultaneously added to the lower chamber for 24 h. After incubation, the cells on the upper surface of the ﬁlter were removed with cotton swabs. Cells invading across the matrigel to the lower surface of the membrane were ﬁxed with 4% formaldehyde, stained with 0.1% crystal violet (in 20% ethanol) and counted with light microscope under 400× magnification. Each experiment was carried out in triplicate.

### 3.6. Orthotopic Tumor Model

Nude mice were injected with 1 × 10^7^ cells (1 × 10^7^ cells/0.2 mL PBS) into the right flank. Once the tumor reached a size of 1 to 1.5 cm in diameter, the mouse was sacrificed. The tumor was resected under aseptic conditions and was cut into about 1 mm^3^ sections which were implanted into the left liver lobe of the nude mice using a 12-gauge trochar [27]. The experiments comprised 32 nude mice, which were randomly devided into four groups. Treatment was started on the third day of orthotopic implantation. Mice were treated either with 0.9% sodium chloride, 50 or 500 μg/kg P-5m five times a week, or 15 mg/kg 5-fluorouracil (5-Fu) upon i.p. administration three times a week. The general physical state of mice were monitored daily. Mice were sacrificed 1 month after starting drug therapy. Every tumor tissue speciment was cut into three pieces for Western blotting, immunohistochemistry and real-time PCR respectively. The main organs, including the liver, lung, heart, spleen, kidney, intestine and sternum were fixed in paraformaldehyde and embedded in paraffin.

### 3.7. Histology and Immunohistochemistry

Serial sections (5 μm) were cut from each block/group and stained with H&E to verify metastasis according to morphologic characteristics. Tumor nodules in the sections were counted in ten randomly selected fields at high power using an Olympus X71 Inverted Microscope [28].

Immunohistochemistry was performed to detect MMP-2 immunoreactivity in sections. A monoclonal antibody against MMP-2 was obtained from AnaSpec (San Jose, CA, USA). Immunoreactivity was detected with UltraSensitive TM S-P kits (Maixin Bio, Fujou, China) according to the manufacturer’s instructions.

### 3.8. Western Blotting

Protein levels of MMP-2 in tumor tissues were also assayed by Western blotting. Tumor tissues from control and experimental mice were homogenized and lysed in ice-cold RIPA lysis buffer containing protease inhibitor. The supernatant protein concentration was measured by the Bradford assay. The protein was separated by SDS-PAGE using a 10% gel and transferred to a PVDF membrane (Bio-Rad). The membrane was blocked with 5% non-fat milk, and then incubated with mouse anti-MMP-2 primary antibody. After washing with TBST, the membrane was incubated with the secondary antibody. An enhanced chemiluminescence kit was used to detect bands. β-actin was chosen as an internal control and the blots were probed with a mouse anti-β-actin mAb (Zhongshan, Inc., Beijing, China). Proteins were quantitatively determined by densitometry using NIH Image software (Image J).

### 3.9. Quantitative Real-Time PCR

Total RNA was extracted from tumor tissue using Trizol reagent (Invitrogen, Gaithersburg, MD, USA) following the manufacturer’s protocol. RNA (1 μg) was reverse transcribed using a First Strand cDNA Synthesis Kit (GE Healthcare, Piscataway, NJ, USA). The real-time PCR was performed with the iQ5 Real Time PCR Detection System (Bio-Rad, Foster City, CA, USA) using SYBR Green I reaction mix (GeneCopoeia, Foster City, CA, USA) and different primers (Table 2). GAPDH gene expression was used as an internal control. A melting curve was generated to conﬁrm the production of a single PCR product. The threshold cycle number (Ct) was calculated for selected genes and GAPDH using the Mx3000P™ Stratagene software. ^Δ^Ct was the difference in the threshold cycles of mRNA for selected genes relative to those of GAPDH mRNA. The n-fold differential ratio was expressed as 2^−ΔΔCt^.

### 3.10. Statistical Analysis

Data are presented as mean ± standard deviation (SD) for the indicated number of independent experiments. Statistical analysis was performed using Student’s *t*-test. A *P*-value of <0.05 was considered statistically significant.

## 4. Conclusions

P-5m, an octapeptide derived from the D5 domain of HKa, showed a significant inhibitory effect on the metastatasis by the HCCLM3 cell line. This effect appeared to occur, at least in part, through regulation of MMP-2 expression. Additional studies are required to further investigate underlying mechanisms including downstream effectors, regulators of metastasis and additional signaling pathways. Further studies will also be needed to investigate the if biochemical modifications to P-5m (e.g., cyclization, conjugation with polyethylene glycol or to albumin) have the potential to prolong the half-time of this moiety and potentially enhance biological potency.

## Supplementary Materials

Supplementary File 1
