# Xiao Han, *et al.*, GHGKHKNK Octapeptide (P-5m) Inhibits Metastasis of HCCLM3 Cell Lines via Regulation of MMP-2 Expression in *in Vitro* and *in Vivo* Studies. *Molecules*, 2012, *17*, 1357-1372

**DOI:** 10.3390/molecules17066996

**Published:** 2012-06-06

**Authors:** Xiao Han, Dong-Mei Yan, Xiang-Feng Zhao, Hiroshi Matsuura, Wei-Guang Ding, Peng Li, Shuang Jiang, Bai-Rong Du, Pei-Ge Du, Xun Zhu

**Affiliations:** 1 Department of Immunology, Norman Bethune College of Medicine, Jilin University, Changchun 130021, China; Email: hanxiaorumeng@126.com (X.H.); yxn345@sohu.com (D.-M.Y.); zxf184@163.com (X.-F.Z.); frank0414@163.com (P.L.); dubairong@yahoo.com.cn (B.-R.D.); 2 Department of Microbial and Biochemical Pharmacy, College of Pharmaceutical Science, Beihua University, Jilin 132001, China; Email: jiangshuang_2000@163.com; 3 Department of Physiology, Shiga University of Medical Science, Shiga 520-2192, Japan; Email: matuurah@belle.shiga-med.ac.jp (H.M.); ding@belle.shiga-med.ac.jp (W.-G.D.)

The authors wish to make the following correction to this paper [1]: the correct name of the fourth author is Hiroshi Matsuura.
