# Supplementary material for: GHGKHKNK Octapeptide (P-5m) Inhibits Metastasis of HCCLM3 Cell Lines via Regulation of MMP-2 Expression in in Vitro and in Vivo Studies
Source: Molecules. 2012 Feb 2;17(2):1357–72. doi: 10.3390/molecules17021357 (PMC6268966; doi:10.3390/molecules17021357)
Supplement: Supplementary File 1 [file molecules-17-01357-s001.pdf]

Molecules **2012**, *17*, 6996; doi:10.3390/molecules17066996

OPEN ACCESS

molecules

ISSN 1420-3049

www.mdpi.com/journal/molecules

Correction

Xiao Han, *et al.*, GHGKHKNK Octapeptide (P-5m) Inhibits Metastasis of HCCLM3 Cell Lines via Regulation of MMP-2 Expression in *in Vitro* and *in Vivo* Studies. *Molecules*, 2012, *17*, 1357-1372

Xiao Han [1,2], Dong-Mei Yan [1], Xiang-Feng Zhao [1], Hiroshi Matsuura [3], Wei-Guang Ding [3], Peng Li [1], Shuang Jiang [2], Bai-Rong Du [1], Pei-Ge Du [2,†,*] and Xun Zhu [1,†,*]

[1] Department of Immunology, Norman Bethune College of Medicine, Jilin University, Changchun 130021, China; E-Mails: hanxiaorumeng@126.com (X.H.); yxn345@sohu.com (D.-M.Y.); zxf184@163.com (X.-F.Z.); frank0414@163.com (P.L.); dubairong@yahoo.com.cn (B.-R.D.)

[2] Department of Microbial and Biochemical Pharmacy, College of Pharmaceutical Science, Beihua University, Jilin 132001, China; E-Mail: jiangshuang_2000@163.com

[3] Department of Physiology, Shiga University of Medical Science, Shiga 520-2192, Japan; E-Mails: matuurah@belle.shiga-med.ac.jp (H.M.); ding@belle.shiga-med.ac.jp (W.-G.D.)

† These authors contributed equally to this work.

* Authors to whom correspondence should be addressed; E-Mails: dupeige2001@126.com (P.-G.D.); zxunzhux@vip.sohu.com (X.Z.); Tel.: +86-431-8561-9476.

Received: 12 March 2012; in revised form: 12 March 2012 / Accepted: 14 March 2012 / Published: 6 June 2012

The authors wish to make the following correction to this paper [1]: the correct name of the fourth author is Hiroshi Matsuura.

Reference

1. Han, X.; Yan, D.-M.; Zhao, X.-F.; Hiroshi, M.; Ding, W.-G.; Li, P.; Jiang, S.; Du, B.-R.; Du, P.-G.; Zhu, X. GHGKHKNK Octapeptide (P-5m) Inhibits Metastasis of HCCLM3 Cell Lines via Regulation of MMP-2 Expression in *in Vitro* and *in Vivo* Studies. *Molecules* **2012**, *17*, 1357–1372.

© 2012 by the authors; licensee MDPI, Basel, Switzerland. This article is an open access article distributed under the terms and conditions of the Creative Commons Attribution license (http://creativecommons.org/licenses/by/3.0/).
